# hiPS-MSCs differentiation towards fibroblasts on a 3D ECM mimicking scaffold

**DOI:** 10.1038/srep08480

**Published:** 2015-02-16

**Authors:** Ruodan Xu, Mehmet Berat Taskin, Marina Rubert, Dror Seliktar, Flemming Besenbacher, Menglin Chen

**Affiliations:** 1 interdisciplinary Nanoscience Center (iNANO), Aarhus University, Gustav Wieds Vej 14, 8000 Aarhus C, Denmark; 2 Faculty of Biomedical Engineering, Technion-Israel Institute of Technology, Haifa 32000, Israel; 3 The Russell Berrie Nanotechnology Institute, Technion-Israel Institute of Technology, Haifa 32000, Israel

## Abstract

Fibroblasts are ubiquitous cells that constitute the stroma of virtually all tissues and play vital roles in homeostasis. The poor innate healing capacity of fibroblastic tissues is attributed to the scarcity of fibroblasts as collagen-producing cells. In this study, we have developed a functional ECM mimicking scaffold that is capable to supply spatial allocation of stem cells as well as anchorage and storage of growth factors (GFs) to direct stem cells differentiate towards fibroblasts. Electrospun PCL fibers were embedded in a PEG-fibrinogen (PF) hydrogel, which was infiltrated with connective tissue growth factor (CTGF) to form the 3D nanocomposite PFP-C. The human induced pluripotent stem cells derived mesenchymal stem cells (hiPS-MSCs) with an advance in growth over adult MSCs were applied to validate the fibrogenic capacity of the 3D nanocomposite scaffold. The PFP-C scaffold was found not only biocompatible with the hiPS-MSCs, but also presented intriguingly strong fibroblastic commitments, to an extent comparable to the positive control, tissue culture plastic surfaces (TCP) timely refreshed with 100% CTGF. The novel scaffold presented not only biomimetic ECM nanostructures for homing stem cells, but also sufficient cell-approachable bio-signaling cues, which may synergistically facilitate the control of stem cell fates for regenerative therapies.

Fibroblasts are ubiquitous cells that constitute the stroma of virtually all tissues, and they are also parenchymal cells in several specialized connective tissues, such as ligaments and tendons^1^,^2^. The poor innate healing capacity of fibroblastic tissues, such as pelvic floor connective tissues, is attributed to the scarcity of fibroblasts as collagen-producing cells^3^.

Fibroblasts are remarkably diverse in different tissues across the body, and even within the same tissue^4^. This diversity is attributed to their poorly-defined origin, in contrast to well-explored induction and differentiation pathways of other mesenchymal lineages, such as osteoblasts, adipocytes, chondrocytes, and myocytes^5^. A longstanding hypothesis of the mesenchymal origin of fibroblasts was proposed with important clues by the discovery of multipotent mesenchymal cells in tendons^6^,^7^. The hypothesis was first proven by a comprehensive study of Lee et al^8^, where connective tissue growth factor (CTGF; also known as CCN2), a 36–38 kDa, cysteine-rich protein of the CCN family^9^ was identified to sufficiently promote MSCs to differentiate into fibroblasts.

Stem cells play important roles in the maintenance and regeneration of terminal differentiated lineages, and thus have been intensively researched as the most promising cell based therapy. Although there are many clinical applications of autologous transplantation of mesenchymal stem cells (MSCs) derived from adult tissues, such as bone marrow and adipose tissue^10^,^11^, their efficacy are hampered by their limited proliferating capacity, which decreases with increasing donor age^12^. In 2006, Shinya Yamanaka made a groundbreaking discovery to ‘reprogramme’ differentiated somatic cells into a pluripotent state, named as induced pluripotent stem cells (iPSCs)^13^, which are capable of differentiating to cell types of all three germ layers^14^,^15^. The iPSC technology has been advanced since then with improved methods for clinically compliant applications^16^,^17^. Among them, MSCs derived from mouse or human iPSCs (iPS-MSCs) have been reported by several groups^18^. Besides the nearly-same multi-potency from MSCs^19^, greater capacity of cell proliferation than bone marrow-derived MSCs^20^ have been seen.

In order to maximize the effectiveness of stem cell based therapies, it is essential to understand the environmental (niche) signals that regulate stem cell behavior. As a key component of the stem cell niche, extracellular matrix (ECM) is a dynamic and complex environment that modulates the maintenance, proliferation, self-renewal and differentiation of the stem cells^21^,^22^,^23^.

Soft connective tissue ECM is a framework of fibrillar collagens with the interstitial space filled with gels of proteoglycans, which not only physically supports the cells and acts as a compression buffer against the external stress, but also contains numerous instructive signals critical for tissue development, homeostasis, and repair^24^.

Both electrospun fibers and hydrogels^25^,^26^,^27^ have been extensively applied as ECM mimicking scaffolds, attributing to their resemblance to the fibrillar framework and the hydrophilic 3D network features, respectively. Looking ahead, it will be interesting to couple the individual microenvironmental cues into more complex substrates that may resemble the *in vivo* situation more closely^28^.

Poly(ε-caprolactone) (PCL), a synthetic, biocompatible polymer that has been approved by FDA as implants, drug delivery devices, sutures, has been applied for a wide variety of applications in tissue engineering research including bone/cartilage, cardiovascular, nerve, skin, tendon/ligament and liver^29^,^30^,^31^. Fibrinogen, on the other hand, as the main coagulation protein in blood, which can easily interact with damaged tissues and cells through native biochemical interactions, has been widely used to construct functional tissues^26^.

Here we construct a novel ECM-mimicking scaffold for fibroblastic commitment of hiPS-MSCs. The semi-synthetic hydrogel precursor, composed of a fibrinogen backbone and poly(ethylene glycol) (PEG) diacrylate (DA) as UV-crosslinkable modules, was infiltrated into the electrospun PCL fibers together with CTGF. Upon exposure to long wavelength UV light which initiates crosslinking between DAs, a 3D ECM-mimicking fibrogenic scaffold was obtained. After biocompatibility evaluations by Lactate Dehydrogenase (LDH) and mitochondrial activity CCK-8 assay, the fibrogenic induction of the scaffold on hiPS-MSCs was assayed by gene expression level through qPCR quantification, protein expression level by immunostaining and flow cytometry.

## Results

### Fabrication of 3D ECM-mimicking nanocomposite matrix

**Figure 1:**
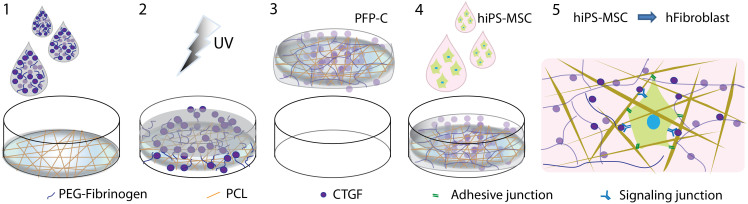
Schematic diagram of preparation of PFP-C nanocomposites and their fibrogenic capacity on hiPS-MSCs. (1) a PF solution with CTGF was added on the top of a PCL mesh; (2) UV light exposure to crosslink PF; (3) a free-standing PFP-C composite was formed; (4) hiPS-MSCs were seeded on the composite; (5) fibrogenesis process synergetically promoted by the adhesive motif on PFP and the signaling induction of CTGF.

**Figure 2:**
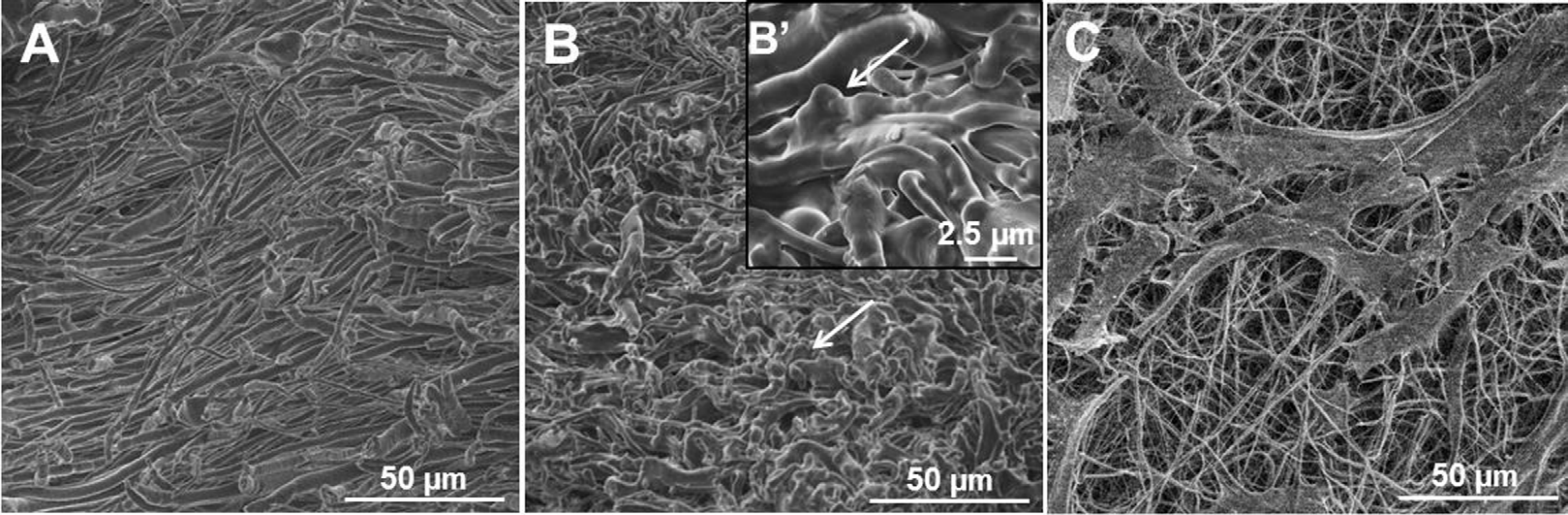
Cross-sectional SEM images of PCL fibers alone (A) and PFP (B, B′) composites, arrows indicate the hydrogel component in the composites.(C) SEM image of hiPS-MSCs seeded on the PFP composite.

The fabrication of the 3D ECM-mimicking fibrogenic scaffold is illustrated in Fig. 1. First, a PCL electrospun mesh which mimics the fibrillar structure of ECM^32^ was placed into a well of 48-well cell culture plate. Cross sectional SEM image showed that the bare electrospun fibers were relatively uniform with fiber diameter of 1.61 ± 0.13 μm. (Fig. 2A). Second, 100 ul of PEGylated fibrinogen (PF) solution was added onto the PCL mesh. Then, cross-linking via DAs upon exposure to UV light^33^ integrated the PCL fibers and PF hydrogel into one 3D scaffold (named PFP). Cross sectional SEM image of the lyophilized sample demonstrated that the interstitial space between the fibers was filled with hydrogel (Fig. 2B).

Although CTGF at concentration of 10 ng/ml was found sufficient to stimulate collagen synthesis of MSCs, 100 ng/ml were most potent^8^. Thus, 40 ng CTGF was suspended in the PF solution before adding on the PCL electrospun mesh, in order to achieve a final concentration of 100 ng/ml CTGF in 400 ul media during cell culture; upon UV exposure, the CTGF loaded fiber/hydrogel composite (named PFP-C) was obtained.

### Biocompatibility

After seeding hiPS-MSCs on the PFP scaffold, the morphology of cells on scaffold was imaged by SEM (Fig. 2C), where the cells and the secreted extracellular matrix (ECM) were found well spread out on the PFP surface.

**Figure 3:**
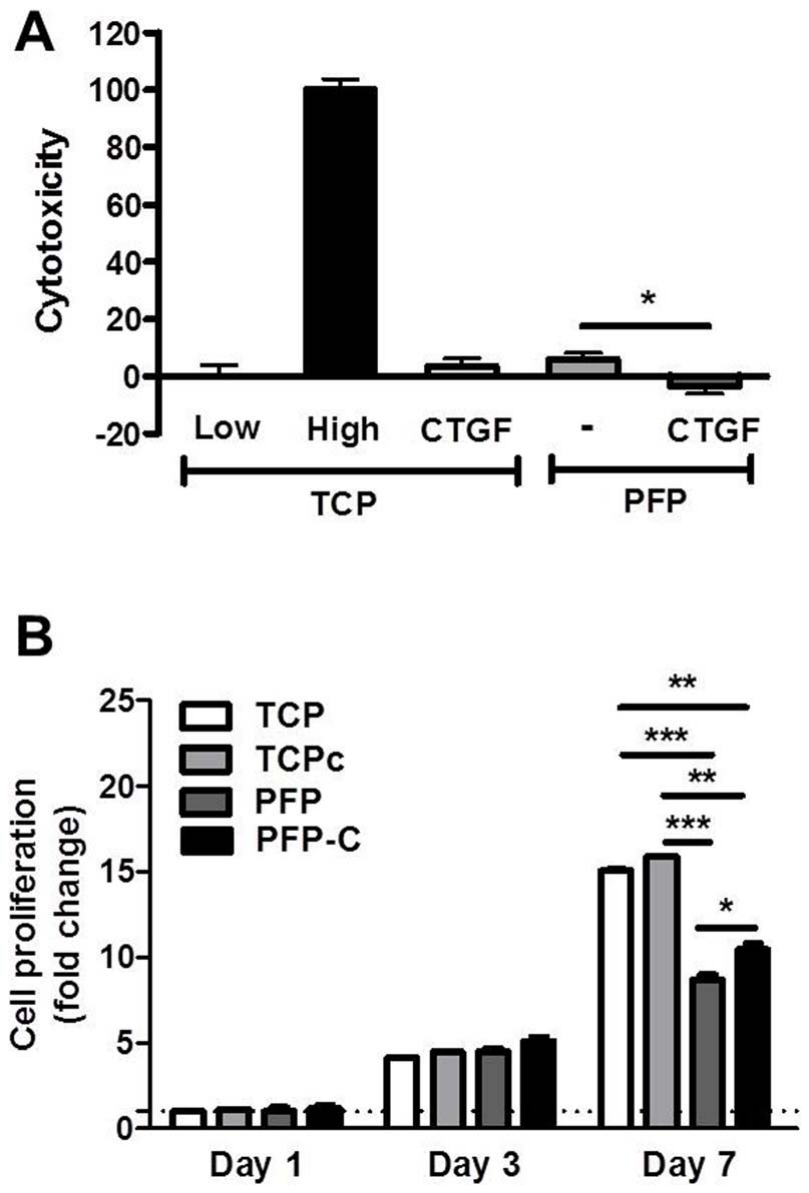
Biocompatibility of nanocomposites PFP and PFP-C with hiPS-MSCs. (A) LDH activity measured from the culture media collected 24 hour after hiPS-MSCs seeding on PFP scaffold and PFP-C scaffold. Low toxicity control (0%) was from cells seeded on tissue culture plastic (TCP). High toxicity control (100%) was from cells seeded on TCP and incubated with 1% Triton X-100. (B) Quantitative analysis of 7 day proliferation indexon TCP, TCPc, PFP and PFP-C scaffolds, data were normalized on day 1 (*P < 0.05, **P < 0.005, ***P < 0.0005).

The Lactate Dehydrogenase (LDH) assay is a reliable colorimetric assay to quantitatively measure lactate dehydrogenase (LDH) released into the media from damaged cells as a marker for cellular cytotoxicity and cytolysis. LDH assay was used after 24 h of culture to assess any cytotoxicity from the PFP and PFP-C scaffolds. As shown in Fig. 3A, both PFP and PFP-C were biocompatible substrates as their LDH releases were measured only 5% of the triton treated cells High control. Notably, the CTGF contained scaffold (PFP-C) further significntly lowered the LDH release, in comparison to the CTGF supplemented 2D TCP cultured cells (named TCPc), demonstrating a superior environment for hiPS-MSCs growth.

Further, to investigate whether the resulting PFP and PFP-C scaffolds support cellular growth of hiPS-MSCs, the mitochondrial activity CCK-8 assay was used to monitor the relative number of viable cells after one week culture (Fig. 3B). Both PFP and PFP-C scaffolds support normal growth of hiPS-MSCs, while the cells on PFP-C proliferated significantly faster than the cells on PFP at day 7. In comparison, no effect from CTGF was observed between TCP and TCPc groups, and the cells grown on TCPs showed a faster proliferation rate on day 7. The different proliferation rates and different effects of CTGF arise from both the 3D architecture and the chemistry nature of the PFP nanocomposites: when the same amounts of the cells were seeded on both TCP groups and PFP groups, cell density on TCP groups was actually lower than that on PFP groups, which might contribute to the lower proliferation rate on PFP groups; the distinct chemistry nature of PFP, compared to tissue culture polystyrene (TCP), provided active interaction with CTGF and overall distinct extracellular environment for the hiPS-MSCs to migrate, proliferate and differentiate.

Both LDH and CCK data suggest the hiPS-MSCs remained viable and were able to proliferate normally on both the PFP and PFP-C scaffolds.

### *In vitro* Release of growth factor CTGF

**Figure 4:**
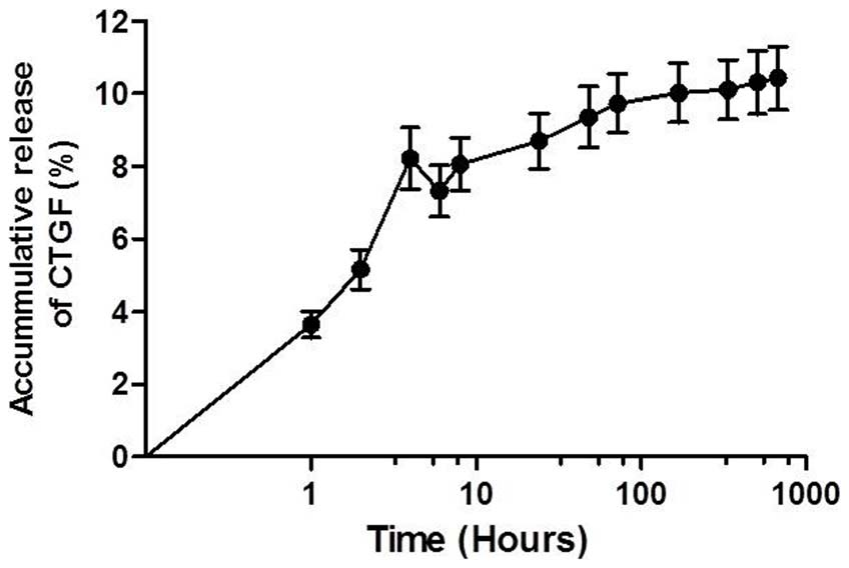
Cumulative release profile of CTGF from PFP-C nanocomposites determined by ELISA assay.

The CTGF released from the PFP-C nanocomposite matrix was determined using ELISA. As shown in Fig. 4, a burst initial release of 8.7% was observed on day 1, followed by a negligible release of 1.7% up to day 28.

### *In vitro* fibrogenesis of hiPS-MSCs in PFP-C

Once the biocompatibility of the 3D nanocomposite scaffold was confirmed, we investigated the fibrogenesis capacity of the PFP-C nanocomposites on hiPS-MSCs, with TCP and TCP supplemented with 100% CTGF refreshment (TCPc) serving as negative and positive controls, respectively. The PFP scaffold without CTGF was also evaluated.

**Figure 5:**
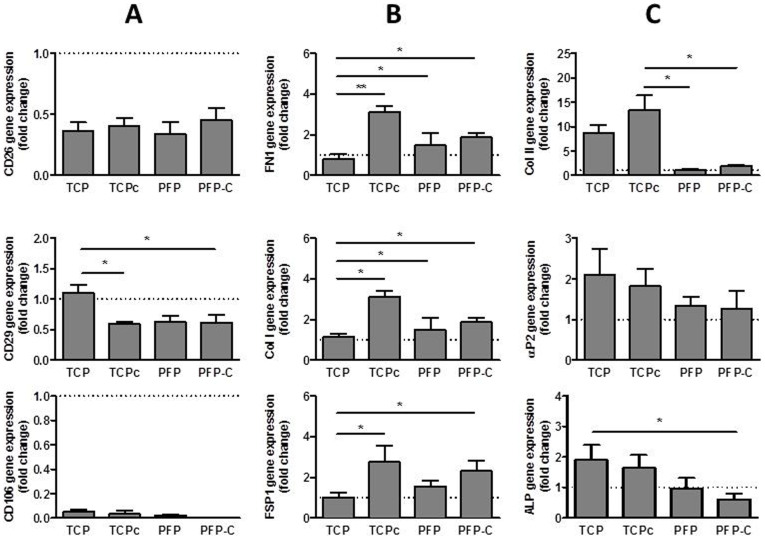
Real time-PCR gene expression analysis of hMSC surface markers CD26, CD29, CD106 (A), fibroblasts markers FSP1, Col I and FN1(B) and adipogenic marker αP2, chondrogenic marker Col II and early osteogenic marker ALP (C) on day 14.(the breaking lines set at 1 are the gene expression levels of undifferentiated hiPS-MSCs) (*P < 0.05, **P < 0.005).

After 2 weeks, the gene expressions of hMSCs surface markers and hallmarkers for fibrogenesis, chondrogenesis, adipogenesis and osteogenesis were all examined by quantitative real-time PCR. The data was summarized as fold changes compared to undifferentiated hiPS-MSCs (Fig. 5).

In comparison to undifferentiated hiPS-MSCs, the expression of hMSCs surface marker CD26, CD29, CD44 (Fig. S1) decreased in all four groups, with significant decreases on CD29 expression in CTGF treated groups (TCPc and PFP-C). In addition, CD106 in all groups became undetectable (Fig. 5A). The attenuated level of surface epitopes indicated that the hiPS-MSCs on all substrates underwent differentiation.

In order to identify the differentiation path, a cascade of fibroblastic hallmarkers, including fibronectin (FN1), collagen I (Col I) and fibroblast specific protein (FSP1)^34^, chondrogenic marker Col II, adipogenic marker αP2, and early osteogeneic marker alkaline phosphatase (ALP) were assayed by real-time PCR.

All fibroblastic markers dramatically increased upon either timely 100 ng/ml CTGF refreshment in the TCPc group, or in the CTGF incorporated PFP-C composite matrix. As shown in Fig. 5B, compared with the cells on TCP, the FN1 gene expression was all significantly up-regulated, with a 2.3 fold increase from the cells in TCPc, a 68% increase in PFP and a 1.1 fold increase in PFP-C; Col I was also significantly increased in all the conditioned groups, with a 1.96 fold increase in TCPc, a 34% increase in PFP and a 73% increase in PFP-C; FSP1 gene expression was significantly increased in both TCPc (1.75 fold increase) and PFP-C (1.3 fold increase), however, no significant differences were found in the PFP group.

Concurrently, adipogenic marker αP2, and osteogeneic marker alkaline phosphate (ALP) were minimally expressed, where ALP expression was significantly decreased in PFP-C compare to TCP group, suggesting adipogenesis and oesteogenesis were both attenuated. While chondrogenic marker Col II was found highly expressed in TCP and TCPc groups, the expression was significantly down-regulated in PFP and PFP-C groups, indicating the chondrogenesis was specificly attenuated in the PFP nanocompsites. (Fig. 5C) Taken all together, real time PCR suggested fibrogenesis of hiPS-MSC in the PFP-C scaffold.

**Figure 6:**
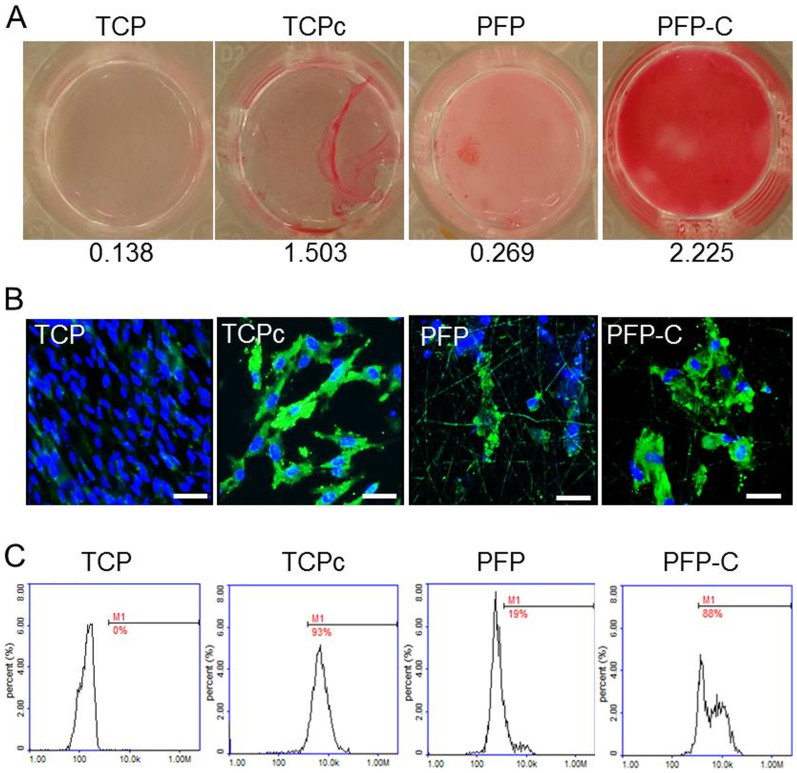
PFP-C nanocomposites enhance hiPS-MSC differentiation towards fibroblasts determined by (A) Collagen content determined by sirius red staining on day 21, (absorbance values at 540 nm are listed) and (B) immunofluorescence analysis of fibroblast marker FSP1(Green) on day 28, nuclei were stained with Hoechst (Blue). Scale bar = 50 μm. Nuclei of TCP group have diameter 4.1 ± 0.22 μm, nuclei of TCPc group have diameter of 6.3 ± 0.23 μm. (C) Flow cytometry quantification of FSP1 expressed cells on day 28.

Consistent with the real-time PCR data, exposure either of 100 ng/ml recombinant human CTGF or the 3D PFP-C composite matrix also induced remarkable collagen synthesis by 3 weeks. As shown in Fig. 6A, collagen staining after colorimetric quantification demonstrated even higher collagen synthesis from PFP-C (Abs_540_ = 2.225) than timely 100 ng/ml CTGF refreshment in group TCPc (Abs_540_ = 1.503), compared to TCP (Abs_540_ = 0.138) and PFP (Abs_540_ = 0.269).

Immunostaining was further applied on evaluating down-stream protein expression levels of the cultured cells after 4 weeks (Fig. 6B). Abundant FSP1 was expressed from the cells on both TCPc and PFP-C scaffolds. Flow cytometry quantification (Fig. 6C) further confirmed that in comparison with the FSP1 expression from the cells on TCP (set as 0%), PFP scaffolds induced 19% FSP1 expression, while PFP-C scaffold promoted FSP1 expression (88%) almost as efficiently as the timely 100% CTGF refreshment condition (TCPc, 93%). Meanwhile, it was noteworthy that the nucleus of the cells exposed to CTGF (TCPc) became significantly larger than the cells without CTGF treatment (TCP) (Fig. S2, Fig. 6B).

## Discussion

Cell therapy based upon stem and progenitor cells have many distinct advantages and offer tremendous potential for regenerative medicine. The multi-potency and proliferative nature of stem cells makes them a more reliable cell source than terminally differentiated phenotypes. Embryonic stem (ES) cells are pluripotent, but their derivation is ethically controversial. Human iPSCs were established^35^ 9 years after the established of human ESCs^36^ which opened up new avenues to generate patient- and disease-specific pluripotent stem cells for advancing regenerative therapy. However, because iPSCs pass through multiple stages of differentiation^37^, implanting iPS cell-derived cells that are too immature may risk yielding mixed populations of undesired cells, or if engrafted when too mature, they might not be adaptable to the surrounding environment. Further, lentiviruses reprogrammed iPSCs may develop into tumors^38^,^39^. Therefore, here we used MSCs derived from mRNA non-integrating reprogramming hiPSC, which shows over 10-fold higher in telomerase activity and greater capacity of cell proliferation than bone marrow derived MSCs^40^, to study their fibrogenesis capacity.

Tissue engineering biomaterial scaffolds are designed to serve as a framework for supporting and allocating cells while providing the appropriate instructive signals for proliferating, regulating and differentiating cells. Rational design of cell-supportive scaffolds has led to the evolution of bio-mimicking scaffolds to replicate the structural and functional hierarchy of the ECM that can interact with and direct stem cell fates, in addition to promoting integration with the host tissue^41^,^42^.

One of the major motivating factors supporting the use of electrospun fibrous scaffolds as supports for stem cell culture is the observation that these fibrous scaffolds mimic the scale and three-dimensional arrangement of collagen fibrils in the ECM^43^,^44^,^45^. Electrospun meshes generally comprise of nonwoven fibers with highly interconnected pores and large surface areas supporting cells spreading^46^,^47^. Also, the ease of functionalization demonstrates their ability to direct MSCs differentiation towards endothelial cells (EC) and smooth muscle cells (SMC)^48^.

Proteoglycan-based three-dimensional (3D) networks composed of cross-linked hydrophilic glycoaminoglycan chains, have been applied in stem cell research and regenerative medicine in recent years^25^. The biological domains in the fibrinogen backbone provide attachment motifs for not only endothelial cells but also epithelial cells adhesion necessary for the development of a blood supply and of newly formed tissue, as well as proteolytic sensitivity to cell-mediated biodegradation. Fibrinogen, acting as a mitogen for tubulointerstitial fibroblasts^49^, has been used for endothelial cell and smooth muscle cell growth and differentiation *in vitro* and *in vivo*^27^,^50^. The semi-synthetic hydrogel composed of a fibrinogen backbone and poly(ethylene glycol) (PEG) diacrylate (DA) UV-crosslinkable modules have been shown to be an excellent matrix for muscle cells^27^. The PEG-fibrinogen (PF) hydrogel was used to make 3D cellularized hydrogel constructs by seeding cells into precursor solution of PF containing a photoinitiator, prior to exposure to long wavelength UV light which initiated crosslinking between DAs and encapsulated the cells without harm^33^. However, a limitation to the use of PF hydrogel is their low mechanical strength and the difficulty to obtain anisotropic structures in order to control the topology of the new tissue.

We appreciate that the natural ECM contains components with different organizational scales in a hydrated state, thus we combined the nano-/micro-scale electrospun fibers which are largely responsible for the structural integrity, with the hydrated, micro-scale porous PF hydrogels which provide functional affinities for stem cells anchorage and GFs storage (Fig. 1,2).

Along with the demonstrated cell viability from LDH and CCK assays (Fig. 3), cell morphology as observed by SEM (Fig. 2C) and confocal imaging (Fig. 6B) demonstrated that the PFP nanocomposite well supported growth of the hiPS-MSCs. With the premise of biocompatibility of our PFP-C scaffolds with hiPS-MSCs, we investigated their fibrogenesis capacity.

Gene expression of a panel of hMSC surface markers together with fibrogenic, oesteogenic, chondrogenic and adipogenic markers were assayed by real time PCR (Fig. 5). Levels of MSC surface epitopes were all attenuated, including CD26, CD29, CD44 and CD106. Concurrently, levels of fibroblastic markers were significantly upregulated, including Col I, fibronectin (FN1) and FSP1 in CTGF treated TCPc and PFP-C groups. Meanwhile, the adipogenic marker αP2, the osteogenic marker OC and ALP and the chondrogenic marker Col II were all significantly suppressed in PFP-C groups. Thus the gene expression clearly demonstrates the fibrogenic capacity of PFP-C on hiPS-MSCs.

PFP-C scaffold promoted collagen I synthesis (Fig. 6A) and FSP1 protein expression (88%) almost as efficiently as the timely 100% CTGF refreshment condition (TCPc, 93%) (Fig. 6B,C). Consistent with gene expression levels (Fig. 5), the PFP-C composites impressively supplied sufficient inductions for differentiating hiPS-MSCs to a fibroblastic lineage, to an extent comparable to the 2D culture with timely 100% refreshment of CTGF (100 ng/ml) conditioned media. The results also suggest that the encapsulation process through photo-crosslinking did not alter at all the structure or function of CTGF.

Further, the *in vitro* CTGF release study showed a 8.7% release of CTGF from the nanocomposites at the initial 24 h after a nearly negligible (1.7%) release over the 28 days (Fig. 4). The initial burst released CTGF probably contributed to reaching those hiPS-MSCs that haven't migrated inside the composites, and stimulating the development of early fibroblast progenitor cells in the very beginning. Approximately 90% CTGF was remained inside the nanocomposite, which maturated the progenitor cells towards fibroblasts during the differentiation progress for 28 days. It suggested PFP functioned as a ECM-mimicking environment for CTGF, which not only bound and stabilized GFs, while GFs in the bulk quickly degraded, but also enriched and clustered GFs to achieve high “local” [GF] to trigger specific signal, while [GF] in the bulk remained low.

In addition, it is noticeable that PFP alone slightly improved fibrogenesis compared with the cells on TCP in 2D, as shown in FSP1 gene and protein expression, providing the hint that PCL/PF composites alone also contributed to the fibroblastic commitment. Collectively, the PFP-C as a fiber-hydrogel-GF nanocomposite, synergistically provided the hiPS-MSCs anchorage, cell mobility, proliferation and differentiation.

From both a fundamental and clinical perspective, our work presented a novel nanocomposite that promoted the active interactions with stem cells and exerted excellent fibrogenic commitment *in vitro*, which should provide instructive insights towards the development of new technologies for stem cell manipulation. Using this method enabled the design of a cell culture construct bearing larger volume to support sufficient amount of cells for *in vivo* implanting in the future. True validation of the capability of the developed nanocomposite on functional tissue regeneration *in vivo* is currently an on-going study in our laboratory.

## Methods

### Preparation of PFP-C

Electrospinning solutions were prepared by dissolving PCL (~80 kDa) (Sigma. Germany)in 8:2 chloroform/ethanol (v/v) into 12% solution (w/v). PCL solutions were electrospun at 20 kV through a 20G blunt end needle with a flow rate of 8 ml/h. Obtained fibers were freezedried overnight. The mesh was punched into circular shapes (12 mm diameter) to fit into 48-well tissue culture plates. The fibers were sterilized by 254 nm UV light for 30 mins before PF hydrogel incorporation.

Poly(ethylene glycol)-Fibrinogen (PF) protein was prepared as previously reported^33^. PF was dissolved together with 0.1% photoinitiator (made of 10% w/v Irgacure®2959 (Irgacure 2959; Ciba Specialty Chemicals, Tarrytown, NY) in 70% ethanol and deionized water in the dark. PCL fibers were pre-wetted by 30 ul ethanol in 48-well plate. The PF solution loaded with or without CTGF (40 ng/well) (Invitrogen, Carlsbad, US) was added on PCL mesh and kept at 4°C overnight before exposure under long-wave UV light (365 nm, 4–5 W/cm^2^) for 10 minutes to perform the cross-linking.

### Scanning electron microscopy (SEM)

The cross-section morphologies of the PFP and the morphology of cells growing on PFP after 48 h incubation were examined with a high-resolution scanning electron microscope (SEM) (FEI, Nova 600 NanoSEM). The fibers were placed directly into the SEM chamber without any metal sputtering or coating.

The hiPS-MSC cells seeded on PFP (after 2 day culture) were fixed with 4% glutaldehyde for 2 hours, washed with water twice, and then dehydrated by the addition of 50%, 70%, 90% and 100% ethanol, removed and evaporate the remaining ethanol before observation. All the images were captured using a secondary electron detector with an acceleration voltage of 5 kV under low vacuum conditions.

### CTGF release

To determine the CTGF release profile from nanocomposites, the PFP-C were immersed in 300 μl of phosphate-buffered saline (PBS, pH7.4) at 37°C and 100 μl of release solution was collected and subsequently refreshed with 100 μl with fresh PBS at 1 h, 2 h, 4 h, 6 h, 8 h and on day 1, 2, 3, 7, 14, 28. The collected specimens were measured using the CTGF Elisa kit according to the manufacturer's protocol (Peprotech, UK). The accumulative release profile was calculated as the mass of released CTGF over time.

### hiPS-MSCs culture and fibroblastic differentiation

hiPS-MSCs generated from human iPS cells as previously reported^40^ were kindly provided by Dr Yonglun Luo, Department of Biomedicine, Aarhus University. hiPS-MSCs were expanded in growth medium of low glucose Dulbecco's Modified Eagle Medium (DMEM-LG, Gibco, UK) containing 10% fetal bovine serum (FBS, Biowhittaker, Walkersvile, MD) and 1% penicillin-streptomycin (Gibco, Grand Island, NY) at 37°C in a humidified atmosphere of 5% CO_2_. Cells were enzymatically treated with Trypsin for passaging every 5–7 days. Passage 5 to 7 of hiPS-MSCs were used for cell differentiation experiment.

Cells were seeded and cultured with growth medium onto each group of samples at a density of 2 × 10^4^ cells cm^−2^ in 48-well plates. At 80–90% cell confluence, iPS-hMSCs were induced to fibroblast differentiation. Fibroblast differentiation was induced in DMEM-LG supplemented with 50 μg/ml ascorbic acid (Sigma Aldrich, Schnelldorf, Germany) and 100 ng/ml CTGF. Cells were seeded on tissue culture plastic (TCP) maintained with medium containing ascorbic acid through the experiment served as negative control whereas a positive control group was maintained by replacing the media with the fibroblast differentiation medium. For the cells seeded on PFP and PFP-C, cells were kept in DMEM-LG medium supplemented only with ascorbic acid. Cells were differentiated for a 2–4 week period, with conditioned medium change every third day.

### Cytotoxicity (lactate dehydrogenase (LDH) activity)

After 24 hours of cell seeding, the LDH activity in the collected culture media was taken as an indicator of damaged cells, sextuplicate of each samples were made under the same conditions. The activity of the cytosolic enzyme was estimated according to the manufacturer's kit instructions (Roche Diagnostics, Mannheim, Germany), by assessing the rate of oxidation of NADH at 490 nm in the presence of pyruvate. After removing the background in the absorbance of the culture media without cells, results from all the samples were presented relative to the LDH activity in the medium of cells treated cultured on TCP (low control, 0% of cell death) and of cells cultured on TCP treated with 1% Triton X-100 (high control, 100% cell death). The percentage of LDH activity was calculated using the equation:
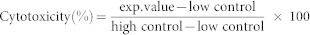


### Cell viability (CCK assay)

Cell viability was assayed with Cell Counting Kit-8 (CCK-8, Dojindo, Kumamoto, Japan). Briefly, cell culture medium were removed, PFP and PFP-C were immersed with diluted CCK-8 solution (30 μl of CCK-8 reagent in 300 μl fresh cell culture medium) in each well and incubated for 2 hours. The same volumes of culture medium and CCK-reagent without cells also incubated as the background. 100 μl of solution was transferred into a 96-well plate, and the absorbance at 450 nm was measured for each well as above. The cell proliferation was examined on days 1, 3 and 7 after incubation, sextuplicate exposures of each samples were made under the same conditions.

### Real-time PCR

Total RNA was isolated using TRIzol (Sigma Aldrich, Schnelldorf, Germany) according to the manufacturer's protocol, triplicate of each samples were made under the same conditions. Resulting total RNA were quantified at 260 nm and 280 nm using a Nanodrop ND-1000 (NanoDrop Technologies, Inc.), and stored at −80°C prior to reverse transcription. 0.8 μg RNA was reverse transcribed to cDNA at 37°C for 60 min using High Capacity RNA-to-cDNA kit (Applied Biosystems, Foster City, CA). For mRNA quantification, real-time quantitative PCR reactions with the cDNA samples were performed in a Lightcycler 480® (Roche Diagnostics, Mannheim, Germany) using SYBR green (Roch) detection with primers listed in Table S1. Fold differences were calculated using the standard ΔΔCt method with GAPDH as the housekeeping. In PCRs with efficiencies approaching 100%, the amount of internal reference gene relative to calibrator (fold change between two Ct values) is given by the equation:
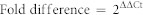


### Quantification of collagen by Sirius Red stain

Total cellular collagen production was quantified at days 21 by staining with 0.1% sirius red solution (0.1% Direct Red 80 in saturated picric acid) (Sigma, UK) for 0.5–1 hr on a platform shaker. The remaining sirius red solution was washed away with deionized water repeatedly until the fluid is colorless. For its quantification, add 200 μl 0.1 M NaOH for 5 min, gently mix by pipetting until the color is eluted from the tissue section, the absorbance of the resulting solution was then measured at 540 nm on a 96-well plate reader.

### Immunostaining and Fluorescence microscopy

Cellular morphology was visualized at day 28 using fluorescence microscopy. Briefly, cells and cell-laden constructs were fixed with 4% paraformaldehyde (PFA) in PBS (pH 7.4) for 15 min at room temperature (RT). After rinsing with PBS for three times, the samples were placed in a permeabilization solution with 0.5% (v/v) Triton X-100 for 15 min and rinsed again with fresh PBS for three times. The cells and constructs were then blocked with 1% BSA in PBS for 1 hour at RT. Immunostaining with primary antibodies mouse monoclonal FSP1 (Abcam, Cambridge, UK) (1:400) and Alexa Fluor 488-conjugated donkey anti-mouse IgG (Life technologies, Carlsbad, CA) (1:1000) was performed at 4°C with gently shaking for overnight, then counterstained with Hoechst 33258 (Life technologies, Carlsbad, CA) to visualize the fibroblasts and nuclei, respectively. Negative control was used with mouse isotype IgG (DAKO, Denmark) antibody. The cells and cell-laden constructs were visualized using a Zeiss LSM 700 laser confocal microscope (Carl Zeiss Micro-Imaging GmbH, Germany).

### Flow cytometry

The number of FSP1 expressing cells was quantified by flow cytometry. Briefly, cells were stripped by trypsin and papain from constructs, resuspended and fixed in 2.5% PFA. After washing in 0.2% Triton, the cells were incubated with primary FSP1 at 4°C overnight. After 400 g centrifugation for 5 mins, the cells were incubated with fluorochrome-labeled secondary antibody Alexa Fluor 488 donkey anti-mouse for 1 hr in the dark and resuspended in PBS, then counterstained with DAPI (ChemoMetec A/S, Allerød, Denmark) at 37°C for 5 min. The samples were then analyzed using NucleoCounter® NC-3000 (ChemoMetec A/S, Allerød, Denmark).

### Statistical analysis

All the experiments were independently repeated 3 times. Data are presented as the means ± standard error of mean (SEM). The statistical analyses were performed using Student's *t*-test or a one-way repeated-measure Analysis of Variance test (ANOVA) to compare multiple data groups, followed by Dunnett's post hoc test. Values of p < 0.05 were considered statistically significant. The software used for statistics and the creation of figures was Prism 5 for Windows (GraphPad, San Diego, CA, USA).

## Author Contributions

M.C. designed the experiments, R.X., M.B.T. and M.R. performed the experiments. R.X. and M.C. wrote the manuscript and prepared the figures. D.S. and F.B. supported the study and provided critical insights and productive discussions. All authors reviewed the manuscript.

## Supplementary Material

Supplementary Information Supplementary materials

## References

[b1] Ronchetti I., Boraldi F., Annovi G., Cianciulli P. & Quaglino D. Fibroblast involvement in soft connective tissue calcification. Front Genet 4, 22 (2013). 23467434 10.3389/fgene.2013.00022 PMC3588566

[b2] Wu M. Regulation of extracellular matrix remodeling associated with pelvic organ prolapse. J Exp Clin Med 2, 6 (2010).

[b3] Van Eijk F. *et al.* Tissue engineering of ligaments: a comparison of bone marrow stromal cells, anterior cruciate ligament, and skin fibroblasts as cell source. Tissue Eng 10, 893–903 (2004). 15265307 10.1089/1076327041348428

[b4] Chang H. Y. *et al.* Diversity, topographic differentiation, and positional memory in human fibroblasts. Proc Natl Acad Sci U S A 99, 12877–82 (2002). 12297622 10.1073/pnas.162488599 PMC130553

[b5] Kwan M. D., Slater B. J., Wan D. C. & Longaker M. T. Cell-based therapies for skeletal regenerative medicine. Hum Mol Genet 17, R93–8 (2008). 18632703 10.1093/hmg/ddn071

[b6] Bi Y. *et al.* Identification of tendon stem/progenitor cells and the role of the extracellular matrix in their niche. Nat Med 13, 1219–27 (2007). 17828274 10.1038/nm1630

[b7] McAnulty R. J. Fibroblasts and myofibroblasts: their source, function and role in disease. Int J Biochem Cell Biol 39, 666–71 (2007). 17196874 10.1016/j.biocel.2006.11.005

[b8] Lee C. H., Shah B., Moioli E. K. & Mao J. J. CTGF directs fibroblast differentiation from human mesenchymal stem/stromal cells and defines connective tissue healing in a rodent injury model. J Clin Invest 120, 3340–9 (2010). 20679726 10.1172/JCI43230 PMC2929735

[b9] Perbal B. CCN proteins: multifunctional signalling regulators. Lancet 363, 62–4 (2004). 14723997 10.1016/S0140-6736(03)15172-0

[b10] Noren Hooten N. & Evans M. K. The ultimate transformers: mesenchymal stem cells. Cell Cycle 10, 4189–90 (2011). 22107963 10.4161/cc.10.24.18490

[b11] Boennelycke M., Gras S. & Lose G. Tissue engineering as a potential alternative or adjunct to surgical reconstruction in treating pelvic organ prolapse. Int Urogynecol J 24, 741–7 (2013). 22940843 10.1007/s00192-012-1927-4

[b12] Minguell J. J., Erices A. & Conget P. Mesenchymal stem cells. Exp Biol Med (Maywood) 226, 507–20 (2001). 11395921 10.1177/153537020122600603

[b13] Bellin M., Marchetto M. C., Gage F. H. & Mummery C. L. Induced pluripotent stem cells: the new patient? Nat Rev Mol Cell Biol 13, 713–26 (2012). 23034453 10.1038/nrm3448

[b14] Takahashi K. & Yamanaka S. Induction of pluripotent stem cells from mouse embryonic and adult fibroblast cultures by defined factors. Cell 126, 663–76 (2006). 16904174 10.1016/j.cell.2006.07.024

[b15] Puri M. C. & Nagy A. Concise review: Embryonic stem cells versus induced pluripotent stem cells: the game is on. Stem Cells 30, 10–4 (2012). 22102565 10.1002/stem.788

[b16] Inoue H., Nagata N., Kurokawa H. & Yamanaka S. iPS cells: a game changer for future medicine. EMBO J 33, 409–17 (2014). 24500035 10.1002/embj.201387098 PMC3989624

[b17] Yoshida Y. & Yamanaka S. Recent stem cell advances: induced pluripotent stem cells for disease modeling and stem cell-based regeneration. Circulation 122, 80–7 (2010). 20606130 10.1161/CIRCULATIONAHA.109.881433

[b18] Teramura T. *et al.* Induction of mesenchymal progenitor cells with chondrogenic property from mouse-induced pluripotent stem cells. Cell Reprogram 12, 249–61 (2010). 20698767 10.1089/cell.2009.0086

[b19] Murphy M. B., Moncivais K. & Caplan A. I. Mesenchymal stem cells: environmentally responsive therapeutics for regenerative medicine. Exp Mol Med 45, e54 (2013). 24232253 10.1038/emm.2013.94 PMC3849579

[b20] Lian Q. *et al.* Functional mesenchymal stem cells derived from human induced pluripotent stem cells attenuate limb ischemia in mice. Circulation 121, 1113–23 (2010). 20176987 10.1161/CIRCULATIONAHA.109.898312

[b21] Gattazzo F., Urciuolo A. & Bonaldo P. Extracellular matrix: a dynamic microenvironment for stem cell niche. Biochim Biophys Acta 1840, 2506–19 (2014). 24418517 10.1016/j.bbagen.2014.01.010 PMC4081568

[b22] Hall P. A. & Watt F. M. Stem cells: the generation and maintenance of cellular diversity. Development 106, 619–33 (1989). 2562658 10.1242/dev.106.4.619

[b23] Watt F. M. & Huck W. T. Role of the extracellular matrix in regulating stem cell fate. Nat Rev Mol Cell Biol 14, 467–73 (2013). 23839578 10.1038/nrm3620

[b24] Badylak S. F., Freytes D. O. & Gilbert T. W. Extracellular matrix as a biological scaffold material: Structure and function. Acta Biomater 5, 1–13 (2009). 18938117 10.1016/j.actbio.2008.09.013

[b25] Seliktar D. Designing cell-compatible hydrogels for biomedical applications. Science 336, 1124–8 (2012). 22654050 10.1126/science.1214804

[b26] Blomback B., Hessel B., Hogg D. & Therkildsen L. A two-step fibrinogen–fibrin transition in blood coagulation. Nature 275, 501–5 (1978). 692730 10.1038/275501a0

[b27] Almany L. & Seliktar D. Biosynthetic hydrogel scaffolds made from fibrinogen and polyethylene glycol for 3D cell cultures. Biomaterials 26, 2467–77 (2005). 15585249 10.1016/j.biomaterials.2004.06.047

[b28] Nosoudi N. *et al.* Engineered Extracellular Matrix: Current Accomplishments and Future Trends. IJBES, 1 **(2)**, 1–15 (2014).

[b29] Ulery B. D., Nair L. S. & Laurencin C. T. Biomedical Applications of Biodegradable Polymers. J Polym Sci B Polym Phys 49, 832–864 (2011). 21769165 10.1002/polb.22259 PMC3136871

[b30] Gunatillake P. A. & Adhikari R. Biodegradable synthetic polymers for tissue engineering. Eur Cell Mater 5, 1–16; discussion 16 (2003). 14562275 10.22203/ecm.v005a01

[b31] Sung H. J., Meredith C., Johnson C. & Galis Z. S. The effect of scaffold degradation rate on three-dimensional cell growth and angiogenesis. Biomaterials 25, 5735–42 (2004). 15147819 10.1016/j.biomaterials.2004.01.066

[b32] Savic R., Luo L., Eisenberg A. & Maysinger D. Micellar nanocontainers distribute to defined cytoplasmic organelles. Science 300, 615–8 (2003). 12714738 10.1126/science.1078192

[b33] Mironi-Harpaz I., Wang D. Y., Venkatraman S. & Seliktar D. Photopolymerization of cell-encapsulating hydrogels: crosslinking efficiency versus cytotoxicity. Acta Biomater 8, 1838–48 (2012). 22285429 10.1016/j.actbio.2011.12.034

[b34] Strutz F. *et al.* Identification and characterization of a fibroblast marker: FSP1. J Cell Biol 130, 393–405 (1995). 7615639 10.1083/jcb.130.2.393 PMC2199940

[b35] Takahashi K. *et al.* Induction of pluripotent stem cells from adult human fibroblasts by defined factors. Cell 131, 861–72 (2007). 18035408 10.1016/j.cell.2007.11.019

[b36] Thomson J. A. *et al.* Embryonic stem cell lines derived from human blastocysts. Science 282, 1145–7 (1998). 9804556 10.1126/science.282.5391.1145

[b37] Galach M. & Utikal J. From skin to the treatment of diseases–the possibilities of iPS cell research in dermatology. Exp Dermatol 20, 523–8 (2011). 21585557 10.1111/j.1600-0625.2011.01282.x

[b38] Yamaguchi S. *et al.* Characterization of common marmoset dysgerminoma-like tumor induced by the lentiviral expression of reprogramming factors. Cancer Sci 105, 402–8 (2014). 24521492 10.1111/cas.12367 PMC4317795

[b39] Okita K., Ichisaka T. & Yamanaka S. Generation of germline-competent induced pluripotent stem cells. Nature 448, 313–7 (2007). 17554338 10.1038/nature05934

[b40] Zou L. *et al.* A simple method for deriving functional MSCs and applied for osteogenesis in 3D scaffolds. Sci Rep 3, 2243 (2013). 23873182 10.1038/srep02243 PMC3718204

[b41] Carletti E., Motta A. & Migliaresi C. Scaffolds for tissue engineering and 3D cell culture. Methods Mol Biol 695, 17–39 (2011). 21042963 10.1007/978-1-60761-984-0_2

[b42] Holzwarth J. M. & Ma P. X. 3D nanofibrous scaffolds for tissue engineering. Journal of Materials Chemistry 21, 10243–10251 (2011).

[b43] Meng J. *et al.* Super-paramagnetic responsive nanofibrous scaffolds under static magnetic field enhance osteogenesis for bone repair in vivo. Sci Rep 3, 2655 (2013). 24030698 10.1038/srep02655 PMC3772377

[b44] Liang Y., Wu D. & Fu R. Carbon microfibers with hierarchical porous structure from electrospun fiber-like natural biopolymer. Sci Rep 3, 1119 (2013). 23350027 10.1038/srep01119 PMC3553486

[b45] Wang X., Ding B., Sun G., Wang M. & Yu J. Electro-spinning/netting: A strategy for the fabrication of three-dimensional polymer nano-fiber/nets. Progress in Materials Science 58, 1173–1243 (2013). 10.1016/j.pmatsci.2013.05.001 PMC7112371 32287484

[b46] Vasita R. & Katti D. S. Nanofibers and their applications in tissue engineering. Int J Nanomedicine 1, 15–30 (2006). 17722259 10.2147/nano.2006.1.1.15 PMC2426767

[b47] Liang D., Hsiao B. S. & Chu B. Functional electrospun nanofibrous scaffolds for biomedical applications. Adv Drug Deliv Rev 59, 1392–412 (2007). 17884240 10.1016/j.addr.2007.04.021 PMC2693708

[b48] Wingate K., Bonani W., Tan Y., Bryant S. J. & Tan W. Compressive elasticity of three-dimensional nanofiber matrix directs mesenchymal stem cell differentiation to vascular cells with endothelial or smooth muscle cell markers. Acta Biomater 8, 1440–9 (2012). 22266031 10.1016/j.actbio.2011.12.032 PMC3289764

[b49] Sorensen I. *et al.* Fibrinogen, acting as a mitogen for tubulointerstitial fibroblasts, promotes renal fibrosis. Kidney Int 80, 1035–44 (2011). 21734641 10.1038/ki.2011.214

[b50] Fuoco C. *et al.* Injectable polyethylene glycol-fibrinogen hydrogel adjuvant improves survival and differentiation of transplanted mesoangioblasts in acute and chronic skeletal-muscle degeneration. Skelet Muscle 2, 24 (2012). 23181356 10.1186/2044-5040-2-24 PMC3579757

