# Supplementary material for: hiPS-MSCs differentiation towards fibroblasts on a 3D ECM mimicking scaffold
Source: Sci Rep. 2015 Feb 16;5:8480. doi: 10.1038/srep08480 (PMC4329554; doi:10.1038/srep08480)
Supplement: Supplementary Information — Supplementary materials [file srep08480-s1.pdf]

Supporting information

hiPS-MSCs differentiation towards fibroblasts on a 3D ECM mimicking scaffold

Ruodan Xu[a], Mehmet Berat Taskin[a], Marina Rubert[a], Dror Seliktar[b,c], Flemming Besenbacher[a], Menglin Chen[a,*]

Table S1

Primers used for RT-PCR

Transcription	Primer sequences	Tm (°C)
CD44	Forward AGCACAATCCAGGCAACTCC	66.5
	Reverse TGTCCCTGTTGTCGAATGGG	68.3
CD106	Forward CTCCTGAGCTTCTCGTGCTC	64.4
	Reverse TGCTTCTACAAGACTATATGACC	63.2
CD26	Forward TGGTCCTGGGATCAGGAAGT	65.9
	Reverse TCTGGAGTTGGGAGACCCAT	65.9
CD29	Forward GCATCCCTGAAAGTCCCAAG	65.8
	Reverse CACTGTCCGCAGACGCACT	68
Col I	Forward CCTGACGCACGGCCAAGAGG	74.5
	Reverse GGCAGGGCTCGGGTTTCCAC	74.1
FN1	Forward CGGAGAGACAGGAGGAAATAGCC	71
	Reverse TTGCTGCTTGCGGGGCTGTC	75
FSP1	Forward AGCTTCTTGGGGAAAAGGAC	63.3
	Reverse CCCCAACCACATCAGAGG	64.2
Col II	Forward GGCAATAGCAGGTTCACGTACA	66.3
	Reverse CGATAACAGTCTTGCCCCACTTA	65.9
αP2	Forward AACCTTAGATGGGGGGTGTC	60.3
	Reverse GTGGAAGTGACGCCTTTC	60.9
ALP	Forward ATGGGATGGGTGTCTCCACA	67.8
	Reverse CCACGAAGGGGAACTTGTC	64.5
GAPDH	Forward TGCACCACCAACTGCTTAGC	65.9
	Reverse GGCATGGACTGTGGTCATGAG	68

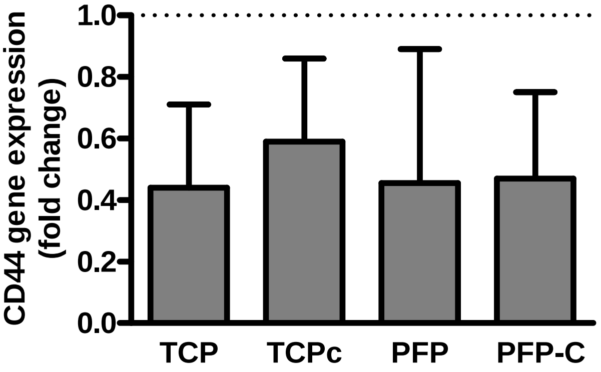

Fig S1. Real time-PCR gene expression analysis of hMSC surface markers CD44 on day 14. (the breaking lines set at 1 are the gene expression levels of undifferentiated hiPS-MSCs)

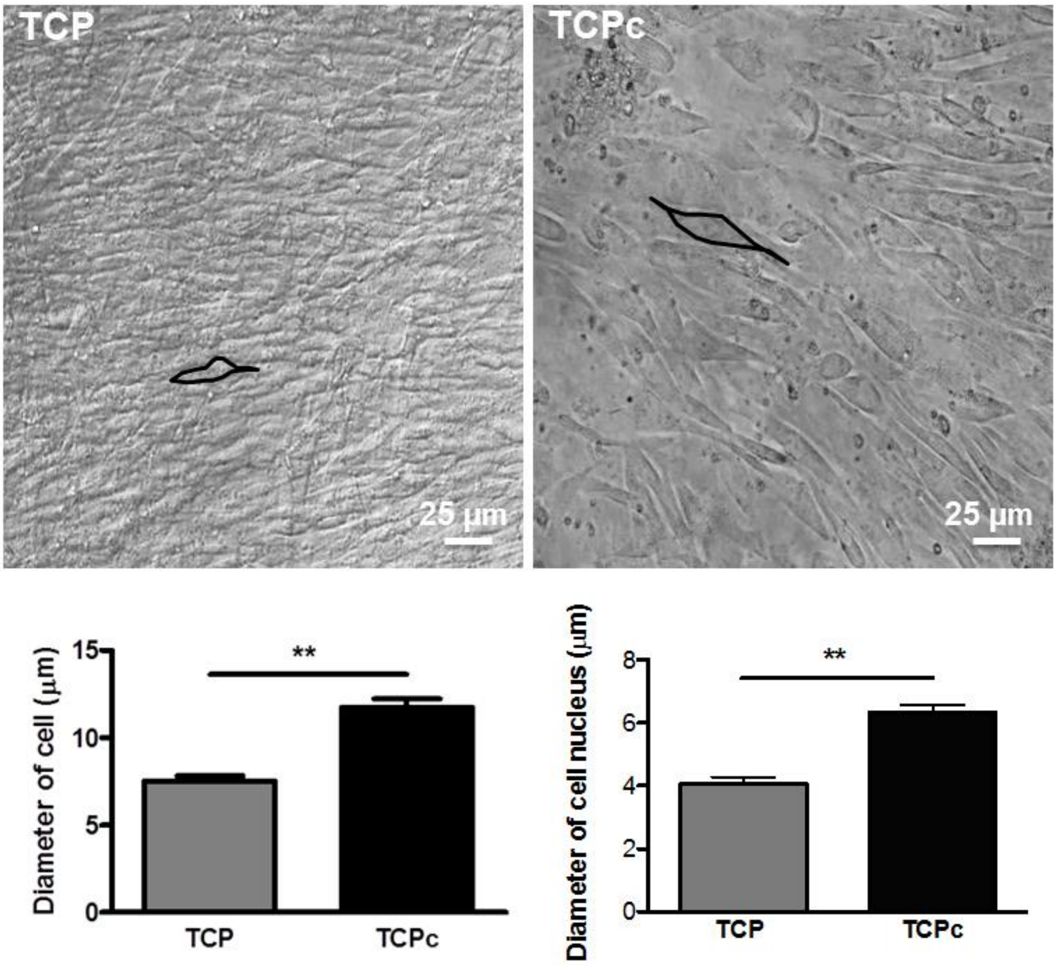

Fig. S2 Light microscopy of hiPS-MSCs on TCP and TCPc after 3 weeks culture. Diameters of cells were measured on 100 cells using imageJ using Fig S1, whereas the diameters of nuclei were measured on 100 cells using ImageJ using Fig 6B. (**$P<0.005$)
